# Combined association of physical activity and sitting time with cardiometabolic risk factors in Chilean adults

**DOI:** 10.1038/s41598-023-36422-8

**Published:** 2023-06-07

**Authors:** Esteban Estrada-Saldaña, Adilson Marques, Danilo R. Silva, Claudio Farías‑Valenzuela, Paloma Ferrero‑Hernández, Juan Guzman-Habinger, Leandro F. M. Rezende, Gerson Ferrari

**Affiliations:** 1 Escuela de Ciencias de la Actividad Física, el Deporte y la Salud, Universidad de Santiago de Chile (USACH), Santiago, Chile; 2 CIPER, Faculdade de Motricidade Humana, Universidade de Lisboa, Lisbon, Portugal; 3 ISAMB, Universidade de Lisboa, Lisbon, Portugal; 4 Department of Physical Education, Federal University of Sergipe, Sao Cristovao, Brazil; 5 Department of Sports and Computer Science, Universidad Pablo de Olavide (UPO), 41013, Seville, Spain; 6 Facultad de Ciencias Para el Cuidado de la Salud, Universidad San Sebastián, Lota 2465, Providencia 7510157 Santiago, Chile; 7 Escuela de Pedagogía en Educación Física, Facultad de Educación, Universidad Autónoma de Chile, 8900000 Santiago, Chile; 8 Sports Medicine and Physical Activity Specialty, Science Faculty, Universidad Mayor, 8580745 Santiago, Chile; 9 Department of Preventive Medicine, Escola Paulista de Medicina, Universidade Federal de São Paulo, Sao Paulo, Brazil

**Keywords:** Diseases, Health care, Risk factors

## Abstract

In this study we examined the combined association of physical activity and sitting time with cardiometabolic risk factors in adults in Chile. This is a cross-sectional study based on 3201 adults aged from 18 to 98 years from the Chilean National Health Survey (2016–2017) who responded to the GPAQ questionnaire. Participants were considered inactive if spent < 600 METs-min/wk^−1^ in physical activity. High sitting time was defined as ≥ 8 h/day. We classified participants into the following 4 groups: active and low sitting time; active and high sitting time; inactive and low sitting time; inactive and high sitting time. The cardiometabolic risk factors considered were metabolic syndrome, body mass index, waist circumference, total cholesterol, and triglycerides. Multivariable logistic regression models were performed. Overall, 16.1% were classified as inactive and high sitting time. Compared to active participants with low sitting time, both inactive participants with low (OR: 1.51; 95% CI 1.10, 1.92) and high sitting time (1.66; 1.10, 2.22) had higher body mass index. Similar results were found for high waist circumference: inactive participants with low (1.57; 1.14, 2.00) and high sitting time (1.84; 1.25, 2.43). We found no combined association of physical activity and sitting time with metabolic syndrome, total cholesterol, and triglycerides. These findings may be useful to inform programs focused on obesity prevention in Chile.

## Introduction

More than 70% of deaths worldwide are caused by noncommunicable diseases^1^. The Latin American region has undergone an accelerated process of epidemiological and nutritional transition, with an increasing prevalence of noncommunicable diseases, such as cardiovascular diseases, cancer, diabetes and respiratory diseases in all age groups from 2005 to 2015^1–3^. Chile’s demographic and epidemiological transitions are among the most advanced in Latin America^4^, and the noncommunicable diseases are considered a major concern as they contribute to 58% of premature deaths in the country^5–7^.

Chile has achieved a high prevalence of overweight/obesity (76%), medium/high cardiovascular risk (55%), and metabolic syndrome (13%)^8,9^, with the latter being defined as a group of cardiometabolic risk factors including abdominal obesity, hypertension, hyperglycemia, and dyslipidemia^10^. This high prevalence of chronic diseases may be explained in part by lifestyle risk factors such as physical inactivity (78% do not meet ≥ 600 METs-min/week^−1^) and high amounts of sitting time throughout the day (84.8% spend > 8 h/day in sitting)^11^.

Physical inactivity and sedentary time have been associated with obesity, metabolic risk, and chronic diseases, such as type-2 diabetes and cardiovascular disease, as well as all-cause mortality^12–15^. Over the last decade, an increasing number of studies hve shown associations of physical inactivity and sedentary time with several health outcomes in children, adolescents, and adults^13,16–18^. A recent study from Chile found no association between meeting both physical activity and sleep duration with cardiometabolic health compared to all three meeting 24-h movement guidelines (e.g., ≥ 600 METs-min/wk^−1^ of physical activity, spend ≤ 8 h/day in sitting time, and obtain between 7 and 9 h/day of sleep duration)^10^. These findings suggest that better health outcomes may be achieved with certain combined behaviors of movement (e.g., high levels of physical activity at any intensity and low sedentary time), but question on whether some intermediate combinations could be better than e.g., low physical activity and low sedentary time for cardiometabolic risk factors—remains uncertain^19–21^. Of note, both in high-income and in low- and middle-income countries, the majority of published research on the relationship between physical activity and sedentary time and health has relied self-reported measures, which are prone to measurement error^22,23^. However, in population-based studies, questionnaires are useful, easy to administer and inexpensive tools, making them well suited to large-scale investigations^24,25^. In this study we examined the combined association between self-reported physical activity and sitting time with several cardiometabolic risk factors in Chilean adults.

## Methods

### Study and sample design

The National Health Survey of Chile (NHS—*Encuesta Nacional de Salud de Chile*) 2016–2017 is a cross-sectional study including a representative sample of residents from different regions of Chile between 15 and 98 years of age, which we have divided in two age categories (adults: 18–64 years; older adults: ≥ 65 years), according to the World Health Organization guidelines^17^.

NHS used a stratified, complex multistage sampling design to select the participants. Thirty strata were considered, which represented urban and rural areas of 15 geographical regions. In the multistage sampling, selection was based on counties as the primary sampling units, households within counties, and finally one participant from selected households using a Kish computational algorithm. Sampling weights from the survey accounted for differences in selection probability and non-response rates, and the post-stratification adjustment allowed to expand the sample to the estimated inhabitants in Chile. Data collection was performed between August 2016 and March 2017. Details of the NHS have been published elsewhere^8,9,11^.

The required sample size was calculated using the absolute sampling error to 2.6% at the national level, 2.5% at the urban national level and 5.9% at the rural national level for a proportion in around 50% to 95% confidence, resulting in a required sample size of 6027. To achieve 6027 interviews, the oversize of the sample response rate for the total sample was 67% based on the NHS 2003–2004 and 2009–2010. Thus, was necessary to consider 10,124 participants with the goal of achieving 6,027 participants.

**Figure 1:**
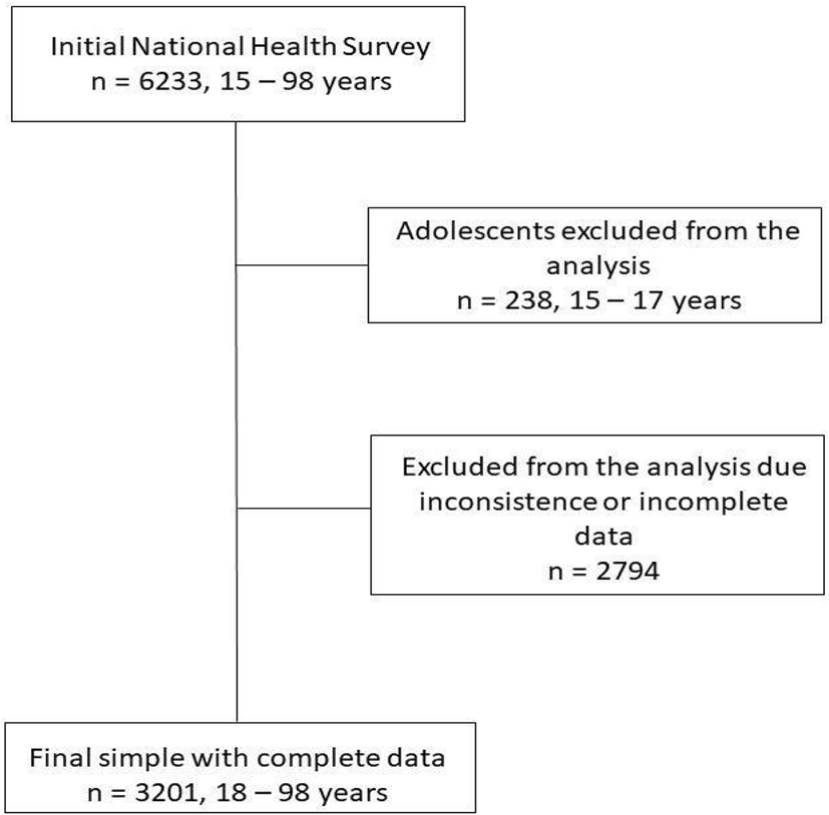
Flow chart of the process to obtain the final sample.

The NHS 2016–2017 included 6,233 participants. For this study, we excluded adolescents aged 15 to 17 years (n = 238) and participants with missing or incomplete information on sociodemographic variables, physical activity, sitting time, and cardiometabolic risk factors (n = 2794). Thus, our final analytical sample included 3201 adult participants (2047 women) aged between 18 and 98 years (Fig. 1).

The NHS was approved by the Ethics Committee of the Faculty of Medicine of the Pontificia Universidad Católica de Chile (No.:16-019). Informed consent was obtained from all subjects and/or their legal guardian(s). All aspects of the study were in accordance with the Declaration of Helsinki and were performed in accordance with relevant guidelines and regulations.

### Assessment of physical activity and sitting time

Self-reported physical activity and sitting time were assessed using the Global Physical Activity Questionnaire (GPAQ), validated internationally^26^ and within the Latin American population^27^. The participants provided information on the duration, frequency, and intensity of the physical activity in three life domains (occupational, transportation, and leisure). Each domain of physical activity was linked to its average Metabolic Energy Equivalents (METs; where 1 METs =  ~ 3,5 ml O2 kg^−1^ min^−1^) according to the GPAQ protocol (4-METs was used for moderate activity and related to active transportation and 8-METs, for vigorous activity). Total self-reported physical activity was calculated as the sum of METs-min/wk^−1^ in the three domains. Participants were classified as physically inactive (< 600 METs-min/wk^−1^) or active (≥ 600 METs-min/wk^−1^)^28^.

Sitting time was evaluated using GPAQ^29,30^ through the following question: (i) “*How much time do you typically spend sitting or lying down at work, at home, going to and from places, or with friends, including time spent sitting at a desk, sitting with friends, traveling by car, bus, or working out, reading, playing cards, or watching television, but does not include time spent sleeping on a typical day?”* Participants responded in hours and minutes per day. This question has shown an acceptable validity, as it has been informed similarly in other countries (r = 0, 23 a 0, 26)^29,30^. Cleland et al. showed a moderate agreement between GPAQ and accelerometer for moderate-to-vigorous physical activity min/day (r = 0.48) and poor agreement for sitting time (r = 0.19)^30^. We applied the cutoff point of ≥ 8 h/day for sitting time, which has been associated with higher risk of cardiovascular diseases and all-cause mortality^20,31^.

Participants were classified according to physical activity and sitting time using the following combined categories: (1) active and low sitting time; (2) active and high sitting time; (3) inactive and low sitting time; (4) inactive and high sitting time.

### Assessment of cardiometabolic risk factors

Cardiometabolic risk factors included metabolic syndrome, body mass index, waist circumference, total cholesterol, and triglycerides.

Metabolic syndrome was defined according to the criteria of the Chilean national guidelines, which requires presenting at least three of the following five criteria: high systolic/diastolic blood pressure (> 135/85 mm/Hg), increased waist circumference (≥ 90 cm in men or ≥ 80 cm in women), elevated total cholesterol (≥ 200 mg/dL), high glycemia (> 100 mg/dL), and elevated triglycerides (> 150 mg/dL)^8,11^.

Height was measured with a portable stadiometer with an accuracy of 0.1 cm. Weight was measured with a digital scale (Tanita HD713) with an accuracy of 0.1 kg. Weight measurements were taken barefoot, and participants wore light clothing^9^. Body mass index (kg/m^2^) was calculated and participants were classified into low weight/normal weight (≤ 24.9 kg/m^2^) or overweight (≥ 25.0 kg/m^2^)^32^.

Waist circumference was measured at the midpoint between the lowest rib and the iliac crest, with a non-deformable plastic band. Measurement was taken on the patient in a standing position and at the end of a normal exhalation. Central obesity was defined as > 88 cm for women and > 102 cm for men^8,33^.

Venous blood samples were obtained after at least 8 h of fasting according to standardized methods described before^8^. Participants with circulating triglycerides ≥ 150 mg/dL or elevated total cholesterol ≥ 200 mg/dL were considered to have elevated triglycerides or cholesterol.

### Covariates

Covariates include sex (male and female), age (adults [18–64 years], and older adults [≥ 65 years]), region (north, center, and south), area of residence (urban and rural), educational level (primary [< 8 years], secondary [8–12 years], and higher education [> 12 years), monthly household income (stratified into terciles: low [< US$ 310.00], medium [US$ 310.00–705. 00] and high [> US$ 705.00]), health insurance (public [Fonasa], private [Isapres] or other/none), indigenous ethnicity (yes and no), smoking (smoker and never/ex-smoker), fruit and vegetable consumption (≤ 4 days/wk and > 4 days/wk), and alcohol consumption. Alcohol consumption was assessed using the short version of the Alcohol Use Disorder Identification Test (AUDIT-C), adapted and validated in Chile, through the following question: “*Have you consumed any drink containing alcohol in the last 12 months?*”, accounted by the categories yes/no, adding also information on frequency and doses each time participant consumed^9,11,34^.

### Statistical analysis

Descriptive data were presented as frequency and proportions according to the combined categories of physical activity and sitting time. Chi-square tests were carried out to compare the differences between combined categories of self-reported physical activity and sitting time (active and low sitting time; active and high sitting time; inactive and low sitting time; inactive and high sitting time) in regard to the sociodemographic characteristics.

We performed multivariable logistic regression models (odds ratio: OR with their respective 95% confidence interval: 95% CI) to estimate the combined association of physical activity and sitting time (independent variable) with cardiometabolic risk factors (dependent variables) adjusted for sex, age, region, area of residence, educational level, monthly income, health insurance, indigenous ethnicity, smoking, fruit and vegetable consumption, and alcohol consumption. We also performed subgroup analysis (both for descriptive and logistic regression) by different age groups (adults and older adults) and sensitivity analysis using a different sitting time cutoffs (6 h/day and 10 h/day). All statistical analyses were performed with SPSS V28 software (SPSS Inc., IBM Corp., Armonk, New York, NY, USA) and accounted for the NHS survey design^8,9,11^. For all tests, a two-tailed *p* < 0.05 was considered indicative of statistical significance.

### Ethics approval and consent to participate

The NHS was funded by the Chilean Ministry of Health and approved by the Research Ethics Committee of the Faculty of Medicine of the Pontificia Universidad Católica de Chile (No. 16-019). All participants gave their written consent before participating. All aspects of the study were in accordance with the Declaration of Helsinki and were performed in accordance with relevant guidelines and regulations.

## Results

A total of 3201 adults with an average age of 50.6 years (standard deviation: 18.3) participated in the study. Overall, 73.9% were between 18 and 64 years of age, 44.5% were smokers, 57.9% consumed fruits and vegetables ≤ 4 days/week and 66.2% consumed alcohol, respectively (Table 1).

**Table 1:** Characteristics of the participants according to combined physical activity and time spent sitting in Chilean adults.

Variables	Total (n = 3201)	Active and low sitting time (n = 990; 31.0%)	Active and high sitting time (n = 222; 6.8%)	Inactive and low sitting time (n = 1475; 46.1%)	Inactive and high sitting time (n = 514; 16.1%)	*p* value
Sex, %						
Men	36.1	34.7	43.7	34.3	40.3	0.024
Women	63.9	65.3	56.3	65.7	59.7	
Age group, %						
Adults (18–64 years)	73.9	72.7	82.4	73.2	74.3	0.006
Older adults (≥ 65 years)	26.1	27.3	17.6	26.8	25.7	
Region of Chile, %						
North	24.9	24.8	31.1	24.1	24.9	< 0.001
Center	26.2	25.3	32.0	23.1	34.6	
South	48.8	49.9	36.9	52.8	40.5	
Geographic area, %						
Urban	84.1	80.8	95.0	81.3	93.9	< 0.001
Rural	15.9	19.2	5.0	18.7	6.2	
Education attainment, %						
< 8 years	24.5	24.8	13.5	27.7	19.5	< 0.001
8–12 years	53.0	56.1	48.6	55.2	43.0	
> 12 years	22.5	19.1	37.8	17.2	37.5	
Monthly household income, %						
Low	48.2	47.8	42.3	51.2	43.0	< 0.001
Middle	36.6	37.8	32.4	36.9	35.2	
High	15.2	14.4	25.3	11.9	21.8	
Health insurance, %						
Public	69.4	70.6	64.9	71.3	64.8	< 0.001
Private	3.7	2.4	7.2	3.0	7.0	
Other/none	26.6	27.0	27.9	25.7	28.2	
Indigenous ethnicity, %						
Yes	11.3	11.4	9.9	11.3	12.1	0.869
No	88.4	88.6	90.1	88.7	87.9	
Tabacco consumption, %						
Smoker	44.5	41.4	47.7	43.6	51.8	< 0.001
Never/former	55.5	58.6	52.3	56.4	48.2	
Fruit and vegetables consumption, %						
≤ 4 days/week	57.9	57.8	66.2	57.1	56.6	0.070
> 4 days/week	42.1	42.2	33.8	42.9	43.3	
Alcohol consumption, %						
Yes	66.2	63.0	73.9	64.9	73.0	0.458
No	33.8	37.0	26.1	35.1	27.0	
Cardiometabolic risk factors						
Metabolic syndrome, %	46.0	46.6	42.8	46.5	44.7	0.025
Overweight, %	77.3	75.3	73.0	79.4	76.8	0.038
Waist circumference above threshold, %	49.0	45.9	42.3	52.7	47.7	< 0.001
High total cholesterol, %	30.8	32.4	27.5	30.6	29.6	0.431
High triglycerides, %	35.0	36.3	35.1	33.9	35.4	0.678

Chi-square tests.

Active: ≥ 600 METs-min/wk^−1^; Inactive: < 600 METs-min/wk^−1^.

Low sitting time: < 8 h/day; high sitting time: ≥ 8 h/day.

Table 1 presents the characteristics of the participants according to combined physical activity and sitting time. Overall, 16.1% were classified as inactive and high sitting time. We found statistically significant differences (*p* < 0.05) in the proportion of combined physical activity and sitting time by sex, age group, region of Chile, geographic area, educational level, monthly household income, health insurance, tobacco consumption, metabolic syndrome, body mass index, and waist circumference (Table 1). Descriptive characteristics by age group (adults and older adults) are displayed in Supplementary Material: Table S1–S2.

**Figure 2:**
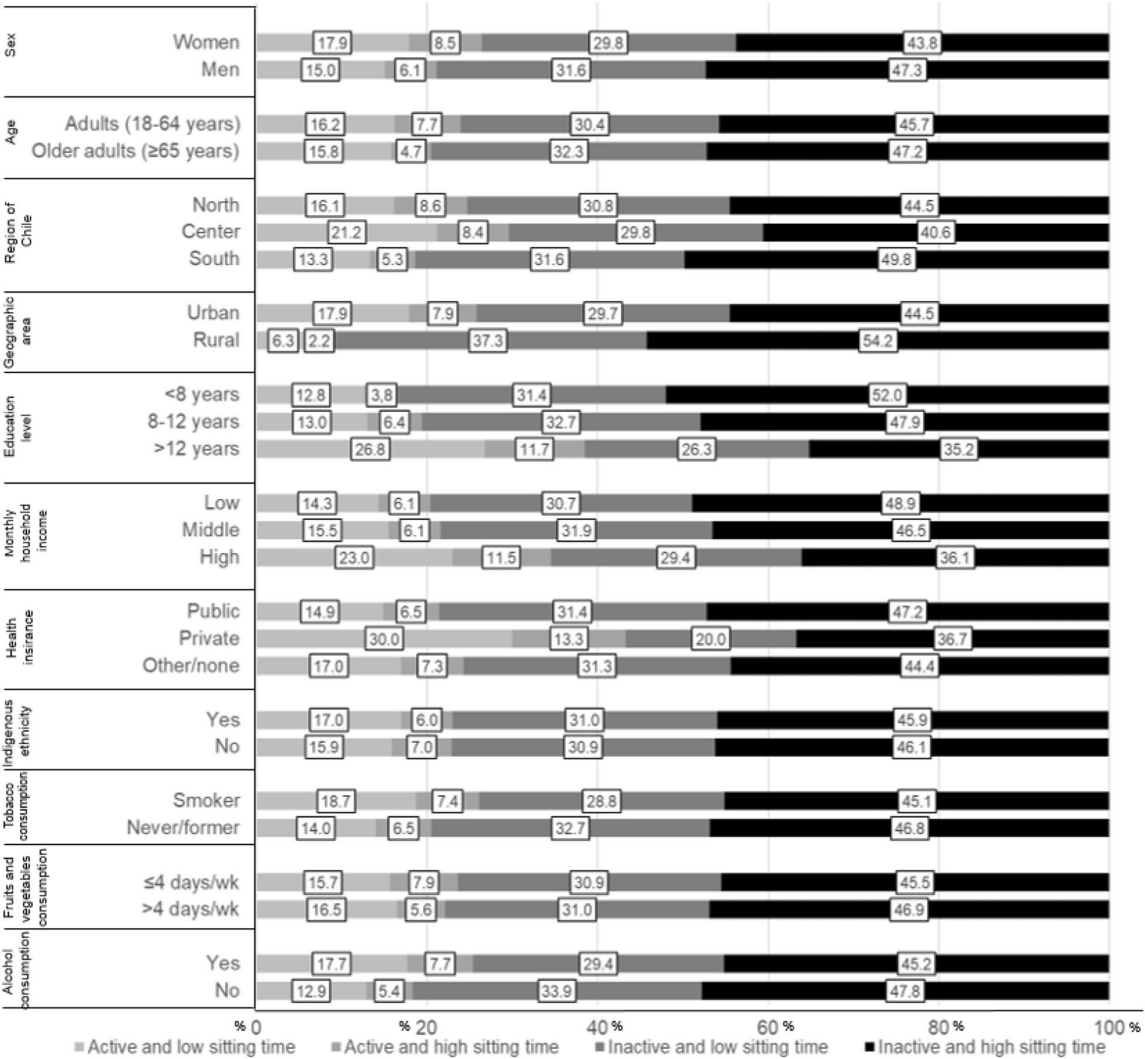
Prevalence (%) of physical activity and sitting time according to sociodemographic and lifestyle variables in Chilean adults. Active: ≥ 600 METs-min/wk^−1^; Inactive: < 600 METs-min/wk^−1^. Low sitting time: < 8 h/day; high sitting time: ≥ 8 h/day.

**Figure 3:**
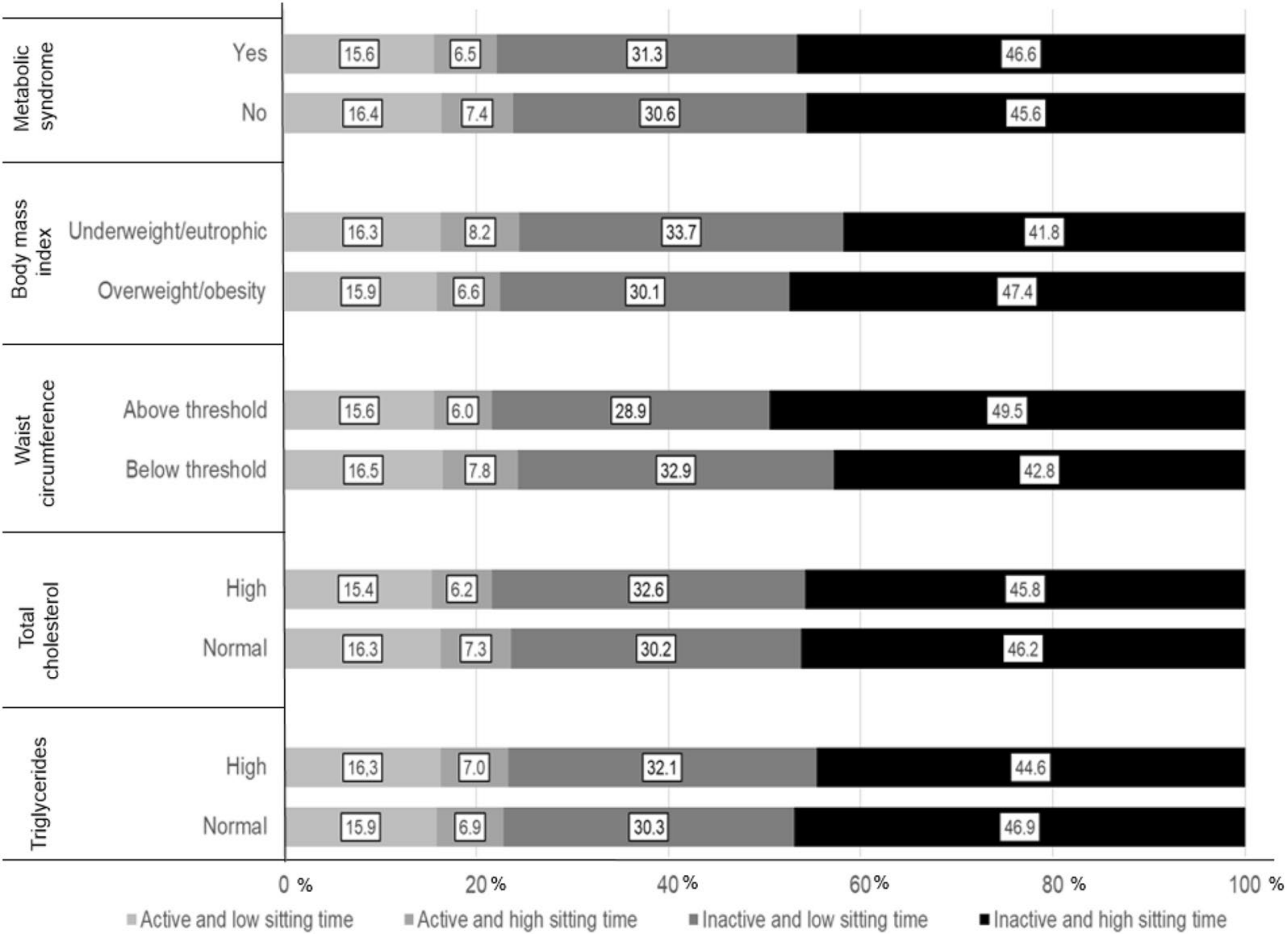
Prevalence (%) of physical activity and sitting time according to cardiometabolic risk factors in Chilean adults. Active: ≥ 600 METs-min/wk^−1^; Inactive: < 600 METs-min/wk^−1^. Low sitting time: < 8 h/day; high sitting time: ≥ 8 h/day.

Figures 2 and 3 show the prevalence of combined physical activity and sitting time according to sociodemographic characteristics and cardiometabolic risk factors. Overall, the prevalence of inactive and high sitting time exceeded 40% in all subgroups analyzed. We found a higher prevalence of physical inactivity and high sitting time in men, adults living in the South and rural areas, those < 8 years of education and low monthly household income, and with access to public health insurance.

We found a combined association of physical activity and sitting time with higher odds of overweight and high waist circumference. Compared to physically active and low sitting time, the OR for overweight were 1.51 (95% CI 1.10; 1.92) for inactive and low sitting time and 1.66 (95% CI 1.10; 2.22) for inactive and high sitting time. Considering the same comparisons, and the OR for high waist circumference were 1.57 (95% CI 1.14, 2.00) for inactive and low sitting time and 1.84 (95% CI 1.25, 2.43) for inactive and high sitting time. We found no combined association of physical activity and sitting time with metabolic syndrome, total cholesterol, and triglycerides (Table 2). Similar results were observed for adults and older adults (Supplementary Material: Table S3). The combined association of physical activity and sitting time (< 6 vs.  ≥ 6, and < 10 vs.  ≥ 10 h/day) with cardiometabolic risk factors were similar compared to the < 8 vs.  ≥ 8 h/day of sitting time (Supplementary Material: Table S4).

**Table 2:** Combined association of physical activity and sitting time with cardiometabolic risk factors in Chilean adults.

Risk factors	OR	95% CI	*p* value
Metabolic syndrome			
Active and low sitting time	1.00		
Active and high sitting time	1.06	0.67; 1.45	0.791
Inactive and low sitting time	1.10	0.84; 1.36	0.463
Inactive and high sitting time	1.21	0.86; 1.56	0.264
Overweight			
Active and low sitting time	1.00		
Active and high sitting time	1.18	0.71; 1.65	0.506
Inactive and low sitting time	1.51	1.10; 1.92	< 0.001
Inactive and high sitting time	1.66	1.10; 2.22	< 0.001
High waist circumference			
Active and low sitting time	1.00		
Active and high sitting time	1.12	0.75; 1.49	0.082
Inactive and low sitting time	1.57	1.14; 2.00	0.006
Inactive and high sitting time	1.84	1.25; 2.43	0.002
High total cholesterol			
Active and low sitting time	1.00		
Active and high sitting time	2.71	0.59; 4.83	0.196
Inactive and low sitting time	2.59	0.85; 4.33	0.092
Inactive and high sitting time	1.25	0.27; 2.23	0.772
High triglycerides			
Active and low sitting time	1.00		
Active and high sitting time	1.06	0.28; 1.84	0.924
Inactive and low sitting time	0.95	0.41; 1.49	0.904
Inactive and high sitting time	1.34	0.52; 2.16	0.542

Active: ≥ 600 METs-min/wk^−1^; Inactive: < 600 METs-min/wk^−1^.

Low sitting time: < 8 h/day; high sitting time: ≥ 8 h/day.

*OR* odds ratio, *95% CI* confidence interval 95%.

*Logistic regression adjusted for sex, age, region, area of residence, educational level, monthly income, health insurance, indigenous ethnicity, smoking, fruit and vegetable consumption, and alcohol consumption in the last twelve months.

## Discussion

The present cross-sectional study examined the combined association of physical activity and sitting time with cardiometabolic risk factors in Chilean adults. We found that participants who were physically inactive, irrespective of high or low sitting time, had higher odds of overweight and high waist circumference. On the other hand, we found no combined association of physical activity and sitting time with metabolic syndrome, total cholesterol, and triglycerides.

Our findings on body mass index and waist circumference are consistent with other studies showing an association between physical activity with lower risk of overweight and obesity^35,36^. In general, these studies do not incorporate the sitting time as an independent risk factor for overweight or obesity. This is especially relevant since it has been previously reported that both physical activity and time spent in sedentary behavior are independent risk factors for increased abdominal adiposity^37^. Contrary to these findings, a study carried out in the Latin American population showed that only moderate-to-vigorous physical activity levels was associated with lower levels of obesity, but not time spent in sedentary behavior^38^.

Previous studies suggest that physical activity may not have an important role on cholesterol levels^39^. This is in agreement with our findings suggesting no combined association between physical activity and sitting time with total cholesterol. On the other hand, triglycerides have been described as an indicator that is more sensitive to modifications with physical activity^40^. In terms of age categories, we found that the association between physical activity and time sitting and cardiometabolic risk factors (metabolic syndrome, body mass index, waist circumference, cholesterol and triglycerides) was similar among adults and older adults.

Various electronic devices have emerged to provide accurate measurements of physical activity levels, one of which is the accelerometer^41^. Previous studies have used accelerometers to establish relationships with cardiometabolic risk factors^42,43^. The study by Silva et al.^42^ has sought to examine the combined association of different intensities of physical activity and sitting time with cardiometabolic risk factors. Their findings suggest no combined associations between the different of physical activity and sitting time with cardiometabolic risk factors. On the other hand, they found associations with compliance of ≥ 150 min/week, even establishing that those people who remained seated for a long time had a lower cardiometabolic risk, compared to those who did not comply with the weekly volume of minutes described. Despite the similarity with our results, they are not comparable, and it is difficult to establish common elements due to the different protocols and assessment instruments used to measure physical activity (accelerometers) and cardiometabolic risk (continuous Metabolic Syndrome score). In addition,, Maddison et al.^44^ suggested that the interrelationships between physical activity and sedentary behavior measured with accelerometers are independent factors on cardiometabolic risk in 10-year projections and must be adjusted to the varied profiles presented by users.

Finally, other methodological issues that may explain the null association with several cardiometabolic risk factors may be related to the assessment of physical activity. Physical activity questionnaires are prone to measurement error. In population-based studies, accelerometers are more valid to measure physical activity and especially sedentary time than questionnaires. Therefore, the association between accelerometer-measured physical activity and health outcomes are stronger than when physical activity is measured with questionnaires^25,45^. This may be due to the differential measurement error or residual confounding associated with self-reported measurements and instruments. Currently, there is insufficient evidence to determine whether, and to what extent, associations between self-reported and device-based assessments of sedentary time differ from health indicators and how they may vary within population subgroups^25,46^. Although physical activity assessment derived from self-report is potentially subject to measurement error, questionnaires are inexpensive tool to measure physical activity, making them well suited to large-scale investigations^24^. Potential confounders considered in our study, such as alcohol consumption, were also self-reported and prone to recall and social desirability biases. However, alcohol biomarkers are more specific and usually not considered in massive health surveys. Moreover, self-reported measure of alcohol consumption demonstrates reliability and validity^47^, likewise, these data collection technique can be improved by incorporating the use of genetic instruments for alcohol consumption, which provide more precisely the causal relationship between alcohol consumption and cardiovascular risk^48^.

The complex temporal and reciprocal relationships between combined physical activity and sitting time with cardiometabolic risk factors remains poorly understood. Countries need to develop these through longitudinal studies to allow greater measurement, surveillance, and promotion of movement behaviors among adults in the Latin America region^11^. These findings are essential for understanding the combined association between physical activity and sitting time with cardiometabolic risk factors in Latin American adults, and therefore establishing evidence-based interventions for preventing cardiometabolic diseases^11^. Prevention should be a top priority for health policy and preventive care should be an indispensable part of the health care system in Chile.

The present study included a representative sample of adults in Chile and adjusted for sociodemographic variables, smoking, fruit and vegetable consumption, and alcohol consumption to examine the combined association of physical activity and sitting time with several cardiometabolic risk factors. However, our study has some limitations. We used self-reported information on physical activity and sitting time, and thus measurement error may have occurred. Previous research has observed differences between self-reported questionnaires and device-measures of physical activity^40^. We used cross-sectional data to examine the association between combined physical activity and sitting time with cardiometabolic risk factors, so there is a possibility of reverse causality and residual confounding.

## Conclusion

We found a combined association between physical inactivity and high sitting time with higher odds of overweight and high waist circumference in adults living in Chile. However, we found no evidence of association with the other cardiometabolic risk factors. These findings may be useful to inform programs focused on obesity prevention in Chile. Future cohort studies are needed to confirm our findings and to examine the association with other cardiometabolic risk factors.

## Supplementary Information

Supplementary Information.

## Data Availability

The datasets generated and/or analyzed during the current study are available in the database repository of the Epidemiology Department of the Chilean Ministry of Health: http://epi.minsal.cl/bases-de-datos/. Data are available upon reasonable request from the corresponding author.
