# Supplementary material for: Combined association of physical activity and sitting time with cardiometabolic risk factors in Chilean adults
Source: Sci Rep. 2023 Jun 7;13:9236. doi: 10.1038/s41598-023-36422-8 (PMC10247732; doi:10.1038/s41598-023-36422-8)
Supplement: Supplementary file 1 — Supplementary Information. [file 41598_2023_36422_MOESM1_ESM.docx]

**Supplementary material**

| Table S1. Characteristics of participants according to the combined categories of physical activity and time spent sitting in Chilean adults (18-64 years). | | | | | | |
| --- | --- | --- | --- | --- | --- | --- |
| Variables | Total  (n = 2365) | Active and low  sitting time  (n = 720; 30.4%) | Active and high sitting time  (n = 183; 7.7%) | Inactive and low  sitting time  (n = 1080; 45.7%) | Inactive and high  sitting time  (n = 382; 16.2%) | p value |
| Sex, % |  |  |  |  |  |  |
| Men | 36.5 | 35.1 | 43.7 | 35.3 | 39.3 | 0.084 |
| Women | 63.5 | 64.9 | 56.3 | 64.7 | 60.7 |  |
| Region of Chile, % |  |  |  |  |  |  |
| North | 26.1 | 26.4 | 32.8 | 24.8 | 26.2 | <0.001 |
| Center | 26.0 | 23.5 | 31.1 | 24.1 | 33.8 |  |
| South | 47.9 | 50.1 | 36.1 | 51.1 | 40.1 |  |
| Geographic area, % |  |  |  |  |  |  |
| Urban | 85.3 | 81.8 | 95.6 | 82.1 | 96.1 | <0.001 |
| Rural | 14.7 | 18.2 | 4.4 | 17.9 | 3.9 |  |
| Education attainment, % |  |  |  |  |  |  |
| <8 years | 13.1 | 13.6 | 5.5 | 15.6 | 8.4 | <0.001 |
| 8-12 years | 60.2 | 64.7 | 49.7 | 63.8 | 46.3 |  |
| >12 years | 26.8 | 21.7 | 44.8 | 20.6 | 45.3 |  |
| Monthly household income, % | |  |  |  |  |  |
| Low | 42.6 | 41.1 | 39.9 | 46.3 | 36.4 | <0.001 |
| Middle | 39.3 | 41.3 | 32.2 | 39.5 | 38.2 |  |
| High | 18.1 | 17.6 | 27.9 | 14.2 | 25.5 |  |
| Health insurance, % |  |  |  |  |  |  |
| Public | 29.9 | 31.0 | 28.4 | 28.7 | 31.9 | 0.002 |
| Private | 4.2 | 2.9 | 8.2 | 3.5 | 6.8 |  |
| Other/none | 65.9 | 66.1 | 63.4 | 67.8 | 61.3 |  |
| Indigenous ethnicity, % |  |  |  |  |  |  |
| Yes | 13.1 | 14.3 | 12.0 | 12.2 | 13.6 | 0.588 |
| No | 86.9 | 85.7 | 88.0 | 87.8 | 86.4 |  |
| Tabacco consumption, % |  |  |  |  |  |  |
| Smoker | 47.5 | 44.2 | 49.7 | 46.8 | 54.7 | 0.008 |
| Never/former | 52.5 | 55.8 | 50.3 | 53.2 | 45.3 |  |
| Fruit and vegetables consumption, % | |  |  |  |  |  |
| ≤4 days/week | 60.1 | 60.6 | 67.8 | 59.5 | 57.3 | 0.116 |
| >4 days/week | 39.9 | 39.4 | 32.2 | 40.5 | 42.7 |  |
| Alcohol consumption, % |  |  |  |  |  |  |
| Yes | 71.1 | 67.9 | 76.0 | 69.9 | 78.0 | 0.002 |
| No | 28.9 | 32.1 | 24.0 | 30.1 | 22.0 |  |
| **Cardiometabolic risk factors** | |  |  |  |  |  |
| Metabolic syndrome, % | 40.4 | 41.1 | 38.3 | 41.1 | 38.0 | 0.034 |
| Overweight, % | 77.3 | 75.7 | 71.6 | 80.2 | 74.6 | 0.011 |
| Waist circumference above threshold, % | 46.4 | 43.8 | 38.8 | 50.4 | 44.0 | 0.003 |
| High total cholesterol, % | 30.7 | 32.1 | 27.9 | 30.1 | 31.2 | 0.668 |
| High triglycerides, % | 34.7 | 34.6 | 32.2 | 34.8 | 35.6 | 0.888 |

Chi-square tests

Active: ≥600 METs-min/wk^-1^; Inactive: <600 METs-min/wk^-1^

Low sitting time: <8 hours/day; high sitting time: ≥8 hours/day

| Table S2. Characteristics of participants according to the combined categories of physical activity and time spent sitting in Chilean older adults (≥65 years). | | | | | | |
| --- | --- | --- | --- | --- | --- | --- |
| Variables | Total  (n = 836) | Active and low  sitting time  (n = 270; 32.3%) | Active and high sitting time  (n = 39; 4.7%) | Inactive and low  sitting time  (n = 395; 47.2%) | Inactive and high  sitting time  (n = 132; 15.8%) | p value |
| Sex, % |  |  |  |  |  |  |
| Men | 34.7 | 33.7 | 43.6 | 31.6 | 43.2 | 0.063 |
| Women | 65.3 | 66.3 | 56.4 | 68.4 | 56.8 |  |
| Region of Chile, % |  |  |  |  |  |  |
| North | 21.5 | 20.7 | 23.1 | 22.0 | 21.2 | 0.003 |
| Center | 26.9 | 30.0 | 35.9 | 20.5 | 37.1 |  |
| South | 51.6 | 49.3 | 41.0 | 57.5 | 41.7 |  |
| Geographic area, % |  |  |  |  |  |  |
| Urban | 80.6 | 78.1 | 92.3 | 79.0 | 87.1 | 0.033 |
| Rural | 19.4 | 21.9 | 7.7 | 21.0 | 12.0 |  |
| Education attainment, % |  |  |  |  |  |  |
| <8 years | 56.8 | 54.8 | 51.3 | 60.5 | 51.5 | 0.093 |
| 8-12 years | 32.9 | 33.0 | 43.6 | 31.6 | 33.3 |  |
| >12 years | 10.3 | 12.2 | 5.1 | 7.8 | 15.2 |  |
| Monthly household income, % | |  |  |  |  |  |
| Low | 64.0 | 65.6 | 53.8 | 64.6 | 62.1 | 0.233 |
| Middle | 28.9 | 28.5 | 33.3 | 29.6 | 26.5 |  |
| High | 7.1 | 5.9 | 12.8 | 5.8 | 11.4 |  |
| Health insurance, % |  |  |  |  |  |  |
| Public | 17.5 | 16.3 | 25.6 | 17.5 | 17.4 | 0.002 |
| Private | 2.4 | 1.1 | 2.6 | 1.5 | 7.6 |  |
| Other/none | 80.1 | 82.6 | 71.8 | 81.0 | 75.0 |  |
| Indigenous ethnicity, % |  |  |  |  |  |  |
| Yes | 6.6 | 3.7 | 0.0 | 8.9 | 7.6 | 0.019 |
| No | 93.4 | 96.3 | 100 | 91.1 | 92.4 |  |
| Tabacco consumption, % |  |  |  |  |  |  |
| Smoker | 36.1 | 34.1 | 38.5 | 34.9 | 43.2 | 0.299 |
| Never/former | 63.9 | 65.9 | 61.5 | 65.1 | 56.8 |  |
| Fruit and vegetables consumption, % | |  |  |  |  |  |
| ≤4 days/week | 51.4 | 50.4 | 59.0 | 50.4 | 54.5 | 0.638 |
| >4 days/week | 48.6 | 49.6 | 41.0 | 49.6 | 45.5 |  |
| Alcohol consumption, % |  |  |  |  |  |  |
| Yes | 52.5 | 50.0 | 64.1 | 51.1 | 58.3 | 0.181 |
| No | 47.5 | 50.0 | 35.9 | 48.9 | 41.7 |  |
| **Cardiometabolic risk factors** | |  |  |  |  |  |
| Metabolic syndrome, % | 61.1 | 64.1 | 61.3 | 64.4 | 61.8 | 0.012 |
| Overweight, % | 77.3 | 74.1 | 79.5 | 77.2 | 83.3 | 0.217 |
| Waist circumference above threshold, % | 56.5 | 51.5 | 59.0 | 59.0 | 5.8.3 | 0.257 |
| High total cholesterol, % | 31.1 | 33.3 | 25.6 | 32.2 | 25.0 | 0.300 |
| High triglycerides, % | 35.8 | 40.7 | 48.7 | 31.4 | 34.8 | 0.028 |

Chi-square tests

Active: ≥600 METs-min/wk^-1^; Inactive: <600 METs-min/wk^-1^

Low sitting time: <8 hours/day; high sitting time: ≥8 hours/day

| Table S3. Combined categories association of physical activity and sitting time with cardiometabolic risk factors in Chilean adults. | | | | | | | | |
| --- | --- | --- | --- | --- | --- | --- | --- | --- |
| Risk factors |  | Adults | | |  | Older adults | | |
|  |  | OR | 95% CI | p value |  | OR | 95% CI | p value |
| Metabolic syndrome | |  |  |  |  |  |  |  |
| Active and low sitting time | | 1.00 |  |  |  | 1.00 |  |  |
| Active and high sitting time | | 1.08 | 0.69; 1.47 | 0.787 |  | 1.05 | 0.67; 1.43 | 0.770 |
| Inactive and low sitting time | | 1.07 | 0.81; 1.33 | 0.459 |  | 1.12 | 0.82; 1.42 | 0.464 |
| Inactive and high sitting time | | 1.19 | 0.84; 1.54 | 0.259 |  | 1.22 | 0.87; 1.55 | 0.260 |
| Overweight | |  |  |  |  |  |  |  |
| Active and low sitting time | | 1.00 |  |  |  | 1.00 |  |  |
| Active and high sitting time | | 1.20 | 0.73; 1.99 | 0.501 |  | 1.17 | 0.70; 1.64 | 0.504 |
| Inactive and low sitting time | | 1.50 | 1.09; 1.91 | <0.001 |  | 1.52 | 1.11; 1.93 | <0.001 |
| Inactive and high sitting time | | 1.67 | 1.11; 2.23 | <0.001 |  | 1.65 | 1.09; 2.21 | <0.001 |
| High waist circumference | |  |  |  |  |  |  |  |
| Active and low sitting time | | 1.00 |  |  |  | 1.00 |  |  |
| Active and high sitting time | | 1.14 | 0.77; 1.51 | 0.080 |  | 1.10 | 0.73; 1.47 | 0.074 |
| Inactive and low sitting time | | 1.53 | 1.10; 1.96 | 0.003 |  | 1.61 | 1.18; 2.04 | <0.001 |
| Inactive and high sitting time | | 1.82 | 1.23; 2.41 | 0.003 |  | 1.86 | 1.27; 2.45 | <0.001 |
| High total cholesterol | |  |  |  |  |  |  |  |
| Active and low sitting time | | 1.00 |  |  |  | 1.00 |  |  |
| Active and high sitting time | | 2.70 | 0.58; 4.83 | 0.186 |  | 2.72 | 0.60; 4.84 | 0.193 |
| Inactive and low sitting time | | 2.58 | 0.89; 4.27 | 0.089 |  | 2.60 | 0.86; 4.34 | 0.088 |
| Inactive and high sitting time | | 1.23 | 0.30; 2.16 | 0.769 |  | 1.24 | 0.26; 2.22 | 0.776 |
| High triglycerides | |  |  |  |  |  |  |  |
| Active and low sitting time | | 1.00 |  |  |  | 1.00 |  |  |
| Active and high sitting time | | 1.03 | 0.25; 1.81 | 0.914 |  | 1.09 | 0.31; 1.87 | 0.910 |
| Inactive and low sitting time | | 0.93 | 0.45; 1.41 | 0.891 |  | 0.95 | 0.41; 1.49 | 0.890 |
| Inactive and high sitting time | | 1.32 | 0.50; 2.14 | 0.539 |  | 1.36 | 0.54; 2.18 | 0.540 |

Active: ≥600 METs-min/wk^-1^; Inactive: <600 METs-min/wk^-1^

Low sitting time: <8 hours/day; high sitting time: ≥8 hours/day

OR: odds ratio; 95%CI: confidence interval 95%

* logistic regression adjusted for sex, age, region, area of residence, educational level, monthly income, health insurance, indigenous ethnicity, smoking, fruit and vegetable consumption, and alcohol consumption in the last twelve months.

| Table S4. Combined categories association of physical activity and sitting time with cardiometabolic risk factors in Chilean adults (n=3201). | | | | | | | | |
| --- | --- | --- | --- | --- | --- | --- | --- | --- |
| Risk factors |  | <6 vs ≥6 hours/day of  sitting time | | |  | <10 vs ≥10 hours/day of sitting time | | |
|  |  | OR | 95% CI | p value |  | OR | 95% CI | p value |
| Metabolic syndrome | |  |  |  |  |  |  |  |
| Active and low sitting time | | 1.00 |  |  |  | 1.00 |  |  |
| Active and high sitting time | | 1.13 | 0.72; 1.54 | 0.456 |  | 1.07 | 0.74; 1.40 | 0.456 |
| Inactive and low sitting time | | 1.10 | 0.83; 1.37 | 0.235 |  | 1.11 | 0.80; 1.42 | 0.456 |
| Inactive and high sitting time | | 1.17 | 0.88; 1.46 | 0.279 |  | 1.15 | 0.90; 1.40 | 0.213 |
| Overweight | |  |  |  |  |  |  |  |
| Active and low sitting time | | 1.00 |  |  |  | 1.00 |  |  |
| Active and high sitting time | | 1.11 | 0.71; 1.51 | 0.201 |  | 1.13 | 0.73; 1.53 | 0.102 |
| Inactive and low sitting time | | 1.34 | 1.04; 1.64 | 0.003 |  | 1.34 | 1.13; 1.55 | 0.004 |
| Inactive and high sitting time | | 1.48 | 1.14; 1.82 | <0.001 |  | 1.39 | 1.13; 1.65 | 0.002 |
| High waist circumference | |  |  |  |  |  |  |  |
| Active and low sitting time | | 1.00 |  |  |  | 1.00 |  |  |
| Active and high sitting time | | 1.19 | 0.88; 1.50 | 0.078 |  | 1.15 | 0.79; 1.51 | 0.067 |
| Inactive and low sitting time | | 1.65 | 1.13; 2.17 | 0.002 |  | 1.78 | 1.21; 2.35 | <0.001 |
| Inactive and high sitting time | | 1.69 | 1.26; 2.12 | <0.001 |  | 1.97 | 1.32; 2.62 | <0.001 |
| High total cholesterol | |  |  |  |  |  |  |  |
| Active and low sitting time | | 1.00 |  |  |  | 1.00 |  |  |
| Active and high sitting time | | 2.23 | 0.60; 3.86 | 0.214 |  | 1.82 | 0.90; 2.74 | 0.093 |
| Inactive and low sitting time | | 2.45 | 0.90; 4.00 | 0.063 |  | 2.23 | 0.83; 3.63 | 0.123 |
| Inactive and high sitting time | | 2.65 | 0.40; 4.90 | 0.645 |  | 2.09 | 0.27; 3.91 | 0.323 |
| High triglycerides | |  |  |  |  |  |  |  |
| Active and low sitting time | | 1.00 |  |  |  | 1.00 |  |  |
| Active and high sitting time | | 1.06 | 0.45; 1.71 | 0.265 |  | 1.11 | 0.34; 1.88 | 0.456 |
| Inactive and low sitting time | | 0.98 | 0.67; 1.29 | 0.090 |  | 0.99 | 0.43; 1.55 | 0.768 |
| Inactive and high sitting time | | 1.45 | 0.89; 2.01 | 0.103 |  | 1.49 | 0.56; 2.42 | 0.678 |

OR: odds ratio; 95%CI: confidence interval 95%

* logistic regression adjusted for sex, age, region, area of residence, educational level, monthly income, health insurance, indigenous ethnicity, smoking, fruit and vegetable consumption, and alcohol consumption in the last twelve months.
